# Effect of Printing Direction of 3D-Printed Nylon Under Abrasive Wear Conditions

**DOI:** 10.3390/polym17212812

**Published:** 2025-10-22

**Authors:** Francisco Briones, Barbara Valverde, Ricardo Donaire, Álvaro González, Federico Antico, Carola Martínez

**Affiliations:** 1 Escuela de Ingeniería Mecánica, Pontificia Universidad Católica de Valparaíso, Valparaíso 2490000, Chile; barbara.valverde@pucv.cl (B.V.); ricardo.donaire.o@mail.pucv.cl (R.D.); alvaro.gonzalez.o@pucv.cl (Á.G.); 2 Facultad de Ingeniería y Ciencias, Universidad Adolfo Ibáñez, Viña del Mar 2562340, Chile; federico.antico@uai.cl; 3 Departamento de Ingeniería en Obras Civiles, Universidad de La Frontera, Temuco 4811230, Chile

**Keywords:** printing orientation, nylon, abrasive wear, thermal modification

## Abstract

This study evaluates the effect of printing orientation on the wear resistance of 3D-printed nylon fabricated via Fused Deposition Modeling (FDM). We conducted abrasive wear resistance tests, thermal analysis, and microstructural characterization using scanning electron microscopy before and after wear testing. The results show that alternating printing directions lead to significantly higher wear. Under a normal load of 130 N, this configuration caused the exposure of up to four layers. At the same time, single-orientation prints exhibit lower material loss and better filament cohesion. DSC analysis reveals that all printed samples, regardless of wear exposure, display dual melting temperatures (T_s1_ and T_s2_) due to distinct crystalline phase formations. Abrasion decreases the secondary melting temperature (T_s2_) and increases enthalpy by up to 144% compared to unprinted nylon, highlighting the thermal history on structural properties. These findings emphasize the critical role of printing configurations in optimizing the tribological performance of 3D-printed nylon for industrial applications.

## 1. Introduction

Additive manufacturing (AM), also known as 3D Printing, has become one of the most innovative manufacturing technologies of recent decades. Its sustainable and versatile approach has allowed its application in fields as diverse as medicine, aerospace, automotive, and architecture [1,2,3,4]. In this context, 3D-printed polymers have emerged as a promising alternative to traditional metal components, thanks to advantages such as the ability to manufacture complex parts quickly and accurately, reduced weight, the creation of a unique product with lower start-up costs, silent operation, and no need for external lubrication [5].

Fused deposition modeling (FDM) stands out among AM technologies for accessibility and versatility. This process heats the polymer above its glass transition temperature and deposits it layer by layer. It enables the fabrication of complex geometries. FDM is compatible with various thermoplastic materials, such as acrylonitrile butadiene styrene (ABS), polylactic acid (PLA), polycarbonate (PC), and polyamide/nylon (PA). These materials offer high chemical resistance and recyclability, which are important for FDM 3D printing. Each material’s unique properties require careful analysis and parameter adjustment to optimize mechanical and functional characteristics [3].

Based on this, numerous studies have been published on the FDM process parameters that can be modified and optimized, such as extrusion temperature, infill density, nozzle diameter, layer thickness, raster angle, and printing direction (infill patterns) [6,7,8]. All these parameters affect the bonding between and within the deposited layers, which in turn modify the material properties. By understanding the influence of these parameters on the printed part, optimal printing parameters can be selected, thereby improving part quality [9]. According to Galeja et al. [10], layer thickness plays a crucial role in mechanical properties, as a higher layer thickness contributes to higher mechanical strength. On the contrary, a lower layer thickness promotes better integration between layers, resulting in smaller cavities and a higher surface quality.

FDM printing has advantages over conventional methods, but surface roughness, low dimensional accuracy, and poor mechanical performance may restrict component performance. Many industries, including automotive, use surface texture modification to address this. Sinmazcelik et al. [11] studied the impact of surface texture formed by 3D Printing on wear. They analyzed two types: microtexture and macrotexture. Their results showed that the wear rate for macrotexture is 28% higher than that for microtexture in the parallel direction (0°). Man et al. [12] created continuous carbon fiber-reinforced polyamide (PA) composites to strengthen parts. Adding fibers reduced the friction coefficient of PA, improved wear resistance, and enhanced its tribological properties.

Zhang et al. [5] focused on evaluating the wear of 3D-printed nylon gears, highlighting the superior performance of nylon compared to other materials, even outperforming injection molded gears under low to medium torque loads. The authors observed distinctive wear patterns, with concentration at the pitch line. Specifically, nylon showed partial melting of the tooth surface without material detachment, in contrast to other materials tested. On the other hand, Portoacă et al. [13] revealed that increasing layer thickness during the Printing of ABS parts correlates with a significant increase in accumulated wear. Specifically, print configurations with a layer thickness of 0.2 mm and a 75% infill percentage resulted in up to seven times higher wear compared to configurations using a layer thickness of 0.1 mm and a 50% infill percentage.

Mahmood et al. [14] investigated the impact of the orientation of printed PLA and ABS layers on their tribological properties. The results indicate that layers printed in a direction orthogonal to the sliding motion exhibit improved wear characteristics, while the coefficient of friction (COF) remains unaffected by the printing orientation. Hanon and Zsidai [15] indicate that the COF for 3D-printed PLA decreases with increasing load when the print layer is oriented at a 45° angle in a vertical position. This phenomenon is attributed to deformation occurring in the contact area. Furthermore, it is observed that the wear rate exhibits an increase at lower loads due to larger gaps between the printed layers, resulting in a reduced contact area. Consequently, the tribological behavior of 3D-printed polymers varies significantly across different materials.

Printing orientation greatly affects the crystallinity and thermal stability of nylon variations. This is mainly due to cooling rates and molecular alignment during layer-by-layer deposition [16]. Higher crystallinity usually improves mechanical strength and thermal stability but can also increase brittleness and reduce adhesion between layers, thereby affecting wear resistance. Lower crystallinity tends to result in a more ductile structure with better energy absorption, but at the cost of lower thermal stability and a higher risk of deformation under load [17]. Nylon has a crystalline structure that exhibits polymorphism [18], making its final properties strongly dependent on the adopted crystalline structure [19]. Optimizing printing parameters is essential in developing new materials through additive manufacturing. It is also crucial to further explore how print orientation affects the abrasive wear resistance of FDM-manufactured nylon. This is important for industrial parts such as gears, bearings, and automotive components that face cyclic loading or constant abrasion. Previous studies have examined infill density, layer thickness, and print speed, but there is little information on how the morphological results of print orientation affect nylon wear.

In this context, this work aims to evaluate the effect of printing orientation on FDM-manufactured nylon parts. This analysis encompasses specific abrasive wear resistance tests, morphological characterization, and thermal analysis, providing practical insights for optimizing component designs in real-world applications.

This study differs from previous research by focusing exclusively on the effect of alternating printing orientations in pure nylon, without reinforcements or changes in infill density. This approach isolates the geometric influence of printing direction on wear mechanisms, providing a reference framework for future studies of nylon-based composites and reinforced polymers.

## 2. Materials and Methods

### 2.1. Manufacturing of Nylon Samples by 3D Printing Process

The samples were manufactured using Taulman 3D Nylon Bridge filaments (Taulman 3D, Saint Peters, MO, USA) with a 1.75 mm diameter. They were manufactured on a Creality Ender 3 3D printer (Shenzhen Creality 3D Technology Co., Shenzhen, China) which is based on FDM technology. Sample designs were created in SolidWorks^®^ 2021 (Dassault Systèmes, Waltham, MA, USA) and saved as printable STL files. The STL file was then converted into G-code using Cura slicing software (Ultimaker B.V., Utrecht, The Netherlands). The G-codes were exported to the FFF machine to print the samples, with printing parameters set to 100% high-quality solid fill.

**Figure 1:**
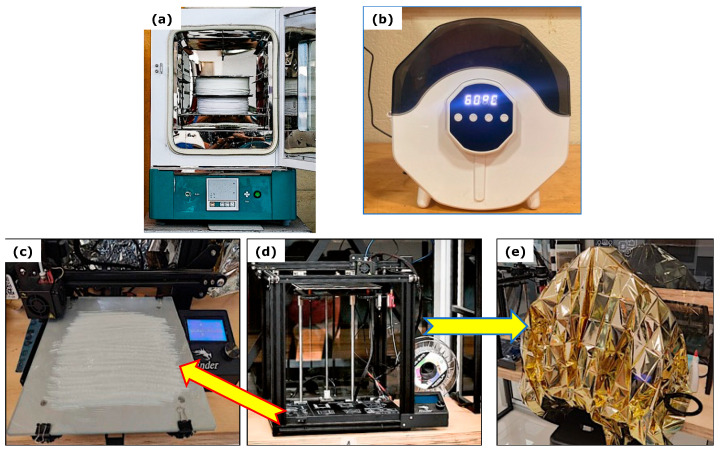
(**a**) Nylon filament drying oven, (**b**) Chamber used to keep the filament dry, (**c**) Tempered glass printing bed coated with PVA solution, (**d**) Creality Ender-3 FDM 3D printer, and (**e**) Thermal insulating blanket used to maintain low humidity and stable temperature conditions during printing.

Before printing, the nylon filament was dried at 60 °C for 8 h to remove moisture (Figure 1a) and then stored in an airtight box to preserve its dry state (Figure 1b) This temperature was selected as it falls within the recommended range (50–70 °C) for nylon pre-drying, ensuring effective moisture removal without thermal degradation [20,21]. Additionally, the printing base was replaced by a tempered glass surface by adding a solution of polyvinyl acetate (PVA) dissolved in distilled water to improve the adhesion between the printed material and the glass surface (Figure 1c). To ensure stable printing conditions and prevent defects such as warping and cracking [22,23], the printer is insulated with a thermal blanket, which protects it from moisture during printing (Figure 1d,e).

The samples were designed with dimensions of 76.2 mm in height, 25.4 mm in width, and 5 mm in thickness. The specimens intended for tribological testing were fabricated using a layer height of 0.2 mm and a nozzle diameter of 0.4 mm. The printing temperature was set at 253 °C, while the build platform was maintained at 70 °C.

Additionally, the main FDM printing parameters included a filament diameter of 1.75 mm, a printing speed of 45 mm/s, a raster width of 0.4 mm, and an extrusion multiplier of 1. The nozzle inner diameter was 1.8 mm.

**Figure 2:**
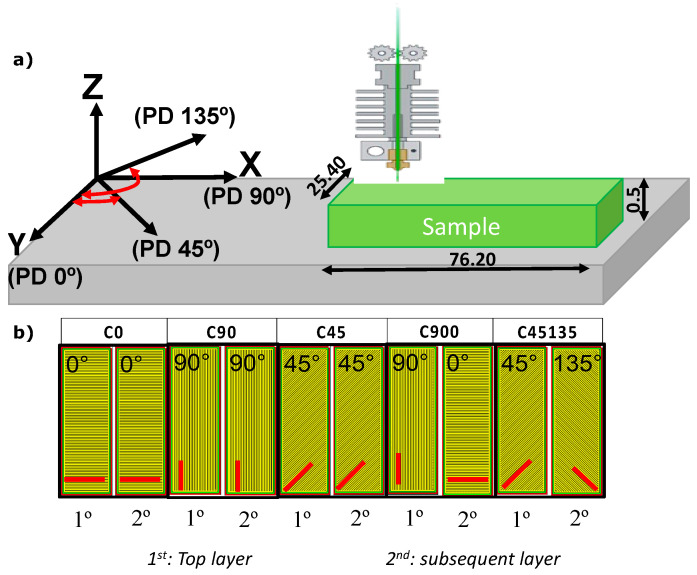
(**a**) Schematic representation of the printing orientations used in the study, showing the reference coordinate system (X–Y–Z) and the corresponding printing directions (PD 0°, PD 45°, PD 90°, and PD 135°) relative to the sample geometry. (**b**) Top (1st) and subsequent (2nd) layer patterns for each sample configuration (C0, C45, C90, C900, and C45135), illustrating the alternating or parallel filament deposition strategy. All illustrations were created by the authors.

Five different printing orientations were used, with all other printing parameters kept constant. Figure 2a shows the five representative printing orientations employed to fabricate the samples. Furthermore, the top and bottom layers, represented in Figure 2b as the 1st and 2nd layers, were fixed at two layers with a repeating pattern during the printing of each sample. For each test condition, five identical samples were prepared.

### 2.2. Wear Tests

**Figure 3:**
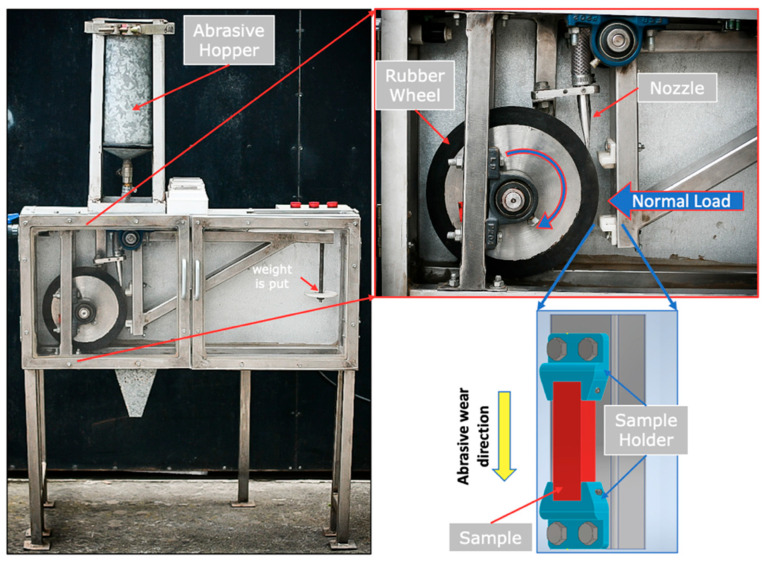
Abrasive wear testing machine setup based on ASTM G65 standard [22].

Abrasion testing was performed using an abrasive wear test based on the ASTM-G65 standard [24], as illustrated in Figure 3. Abrasive particles were fed by gravity through a nozzle, maintaining a continuous abrasive flow of 300 g min^−1^ during testing, falling in a controlled manner at the interface between the vulcanizing wheel and the sample. As the vulcanizing wheel rotates constantly at a speed of 200 rpm, it drags the particles along in its motion, causing them to roll and slide on the surface of the 3D-printed sample.

All tests were performed under dry conditions at 25 °C and 45–50% relative humidity. Each test was performed in triplicate to verify repeatability. Silicon oxide (SiO_2_) abrasive particles with a 50-mesh size (212–300 μm, average diameter 240 μm) were used. Normal loads of 45, 85, and 130 N were applied for 10 min each using calibrated weights. These loads were chosen to produce significant wear but not exceed a volumetric loss of 100 mm^3^ [24]. These load levels (45, 85, and 130 N) are slightly higher than those commonly reported in laboratory tests [25]. They were selected to represent low-, medium-, and high-load regimes. This approach simulates more severe industrial conditions in which the material is subjected to higher contact pressures. This expanded range allowed a clearer distinction between the transitions of different wear micro-mechanisms (micro-plowing and micro-cutting) under different applied loads.

Any unwanted residue was removed from each test. Both the wear testing machine and the samples were cleaned with a cleaning agent (ethanol) to ensure accurate results. The samples were air-blasted and wiped before and after the wear test to remove dirt or dust. The mass of the samples was measured before and after testing using a digital microbalance with a sensitivity of ±0.1 mg. The detailed conditions of the abrasion test are summarized in Table 1.

### 2.3. Characterization of Wear Tracks

The sample surfaces, worn surfaces, and the number of detached layers in the wear track were observed and analyzed using a high-resolution field emission cathode scanning electron microscope (FESEM) (now Thermo Fisher Scientific, Waltham, MA, USA), model Quanta FEG 250 (FEI Company, Hillsboro, OR, USA). The worn areas were quantified using tools such as grayscale conversion (from RGB image to 8-bit), automatic conversion, and thresholding to create binary images, all within ImageJ software version 1.54g. Average 2D linear profiles were taken along the wear track to identify profile depth as a function of normal load and print layers. This procedure is performed using a milling machine table equipped with a vice and parallels to ensure sample flatness. Using a dial gauge calibrated at zero in a non-wear region, the table is moved longitudinally in 0.5 mm increments, recording the depth at each point along the center of the wear track. Therefore, the 2D profiles formed during the abrasion tests were characterized by evaluating the degree of penetration (*D_p_*), which considers the depth of the profiles (*d*) and the radii of the contacts (*a*). The *D_p_*, as initially proposed by Hokkirigawa and Kato [26], is expressed in Equation (1).

(1) $$D_{p}=\frac{d}{a}$$

The parameter *D_p_* is crucial for identifying the severity of wear. Hokkirigawa and Kato [26] suggested a transition in the wear mechanism from micro-ploughing to micro-cutting as the groove depth increases. A similar approach can be applied to understand the wear modes observed in the abrasion samples test results by considering the impact marks as grooves of shorter length and wider width.

### 2.4. Thermal Tests

Differential scanning calorimetry (DSC) and thermogravimetric analysis (TGA) were conducted to evaluate the thermal properties and degradation behavior of the following materials: (i) as-received Nylon, (ii) C0 exposed to abrasion, (iii) C0 not exposed to abrasion, (iv) C45135 exposed to abrasion, and (v) C45135 not exposed to abrasion. DSC experiments were performed using a DSC 300 Caliris Classic system under a controlled atmosphere, with a temperature range of 20 to 300 °C and a heating rate of 10 °C min^−1^. TGA measurements were carried out using a TG 209 F1 Libra system, operating over a temperature range of 20 to 800 °C with a heating rate of 10 °C min^−1^. These analyses aimed to determine changes in melting and thermal decomposition, as well as associated enthalpy changes in the tested samples exposed to the 3D printing process and abrasion. The use of DSC and TGA for characterizing thermal transitions and decomposition in polymers, including Nylon, is well-documented in the literature, as these techniques provide reliable data on melting behavior, crystallization, and thermal stability [27,28,29]. By employing these methods, we aimed to investigate the influence of printing patterns and mechanical stress on the thermal properties of nylon.

## 3. Results

### 3.1. Morphological Analysis of the Samples

**Figure 4:**
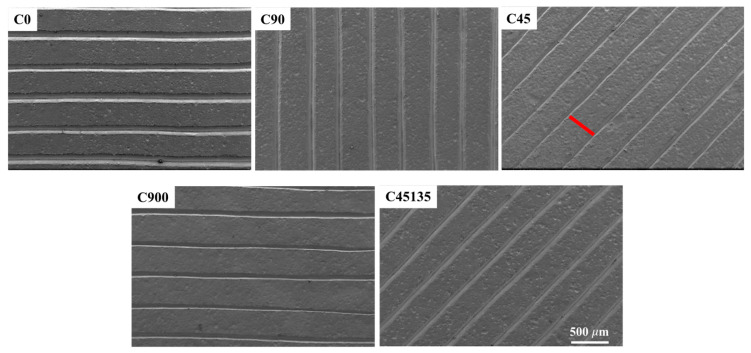
SEM images of the surface morphology of each printed sample.

Figure 4 illustrates the printing directions used for each sample, highlighting the arrangement of the deposited filaments. The fabricated samples revealed details of the printing process, including the periodicity and orientation of the filaments, as well as the absence of significant gaps between them. When observing samples C900 and C45135, the surface layer orientation is identical to that of samples C0 and C45, respectively. This is because, although the printing directions alternate in each subsequent layer, the final surface appearance remains visually similar among these samples.

Variations in the printing directions resulted in differences in the final thickness and surface appearance of the deposited filaments. For sample C45, the maximum thickness variation within the sample reached 5%. When comparing different samples, the maximum difference was approximately 3%, observed between samples C45135 and C0. These variations are intrinsically related to the orientation and distribution of the deposited filaments [30].

**Figure 5:**
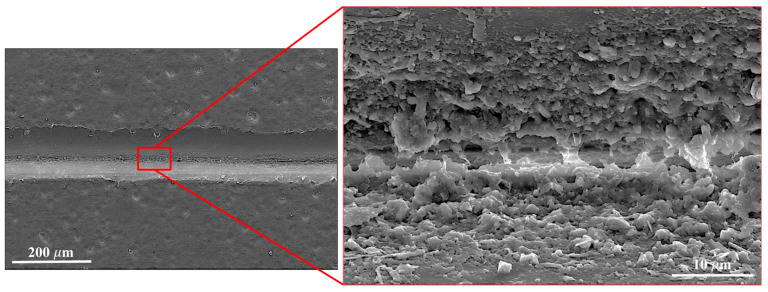
High-magnification SEM images of cohesion between deposited filaments (Sample C0).

SEM analysis of sample C0 reveals the interface between adjacent filaments (Figure 5), showing the bonded area typical of the FDM process. The enlarged view highlights the contact zone, where partial fusion and extruded shape define surface continuity. These FDM-specific features match those reported in previous studies [31].

Variations in print orientation affect layer overlap, which impacts contact area and interlaminar adhesion. The bonding patterns in Figure 5 agree with reports for nylon [32], reinforced nylon [32], and other polymers [33].

### 3.2. Abrasive Wear

**Figure 6:**
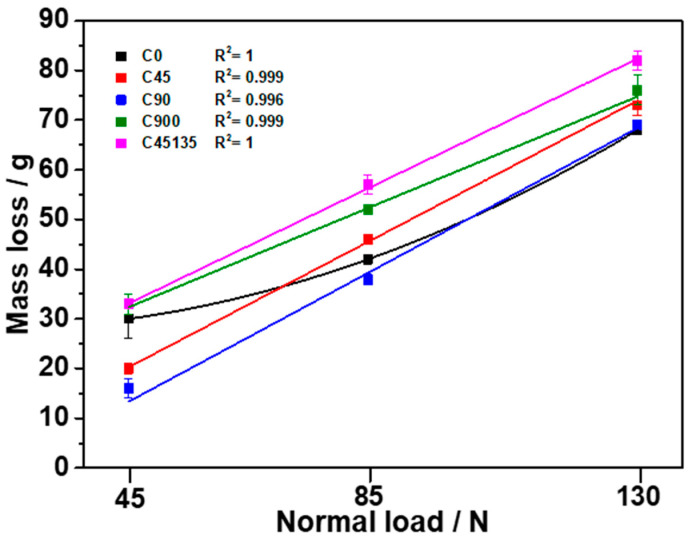
Relationship between mass loss and normal load in printed nylon samples with different printing directions.

Figure 6 presents the abrasion mass loss as a function of normal load, and R^2^ values are indicated for each sample. The abrasion mass loss for all samples increases as the normal load increases. It is important to note that sample C0 has a slightly different situation from the others, as its mass loss follows a quadratic trend (R^2^ = 1). This phenomenon can be attributed to the first contact during local abrasion. According to the studies by Dhakal et al. [34], higher wear track formation was observed at an apparent contact pressure of 5 N compared to 10 N. Furthermore, the authors attribute the increases in initial wear at low loads to the presence of voids and surface irregularities in the 3D-printed material, which significantly influence the initial wear behavior. From the experimental results, it is observed that the mass loss of sample C45135 exhibited the highest mass loss, approximately 52, 31, and 16% at loads of 45, 85, and 130 N, respectively, compared to sample C90, which had the lowest mass loss among the tested samples. However, samples C900 and C45135 are considered to be the samples printed by varying the printing direction per layer, reflecting the highest mass losses. Therefore, these increases in mass loss are consistent with results obtained from other wear studies on printed polymers [35,36], which relate the increased mass loss to the formation of increased voids and weakness in the fusion between layers.

**Figure 7:**
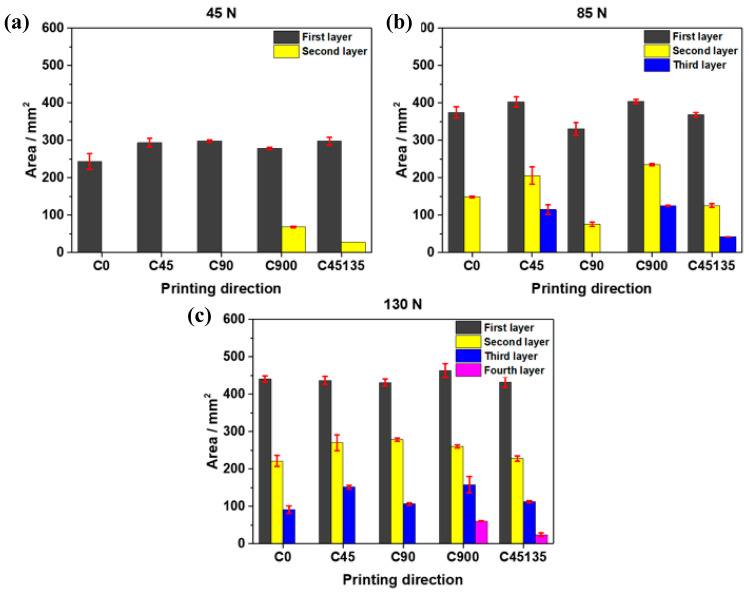
Evolution of the worn area and exposure of internal layers under different normal loads for nylon samples: (**a**) 45 N, (**b**) 85 N, and (**c**) 130 N.

The wear behavior and the progressive exposure of the inner layers of the samples as the applied normal loads increase are shown in Figure 7 for all samples at loads of 45, 85, and 130 N. The results presented include the standard deviation for each condition, allowing visualization of the consistency and repeatability of the measured worn areas under different loads and printing orientations. In general, it can be seen that as the load increases, the worn area and the exposed layers increase for the different printing directions.

When comparing the C900 and C90 samples in terms of total accumulated wear (the sum of all exposed layers), it was observed that at 45 N, the C900 exhibited 27.3% more worn area than the C90. This difference increased to 40.5% under a load of 85 N, indicating a higher susceptibility to wear due to the alternating filament orientation in C900. However, at 130 N, the difference decreased to 15.5%. In this condition, C900 reached the exposure of the fourth layer, whereas C90 was limited to the third layer. This behavior demonstrates that the alternating orientation not only increases the surface wear area but also promotes deeper wear progression, exposing more layers, particularly under intermediate loads [37].

On the other hand, the printing direction directly affects the effective contact area during abrasion tests, which directly influences the wear resistance of the samples. This is associated with the fact that in samples with a single printing direction (such as C0 or C90), the deposited material lines have a uniform orientation, which increases the contact area on the filament surface, between adjacent filaments, and between layers, thereby reducing the presence of weak points. This configuration results in smaller areas and exposure of layers, as observed in sample C90. In contrast, samples with alternating directions (C45135 and C900) exhibit exposure of layers (second layer) to initial loads (45 N), reaching four layers at loads of 130 N (fourth layer), and present more significant variability in the arrangement of the material. This phenomenon increases the interlaminar gaps, which decreases the effective contact area and, therefore, the wear resistance. However, unlike previous studies, which focused on general wear trends in ABS and composite polymers [38,39], these results specifically demonstrate how alternating printing directions in pure nylon samples lead to progressive multi-layer exposure under abrasive loading, highlighting a novel mechanism of layer-by-layer detachment that has not been previously reported for unreinforced nylon.

To complement the graphical results shown in Figure 7, Table 2 summarizes the average worn area and standard deviation for each printing direction under loads of 45, 85, and 130 N.

**Figure 8:**
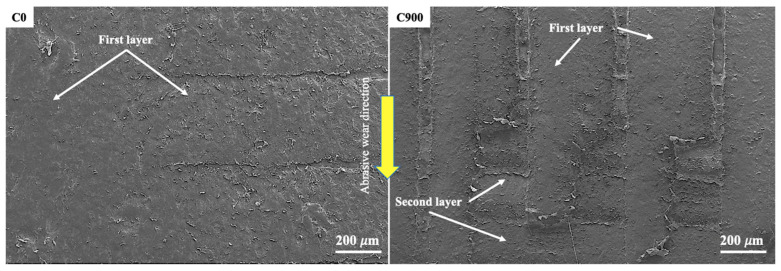
SEM images of samples C0 and C900 show abrasive wear in an enlarged region of the total worn area. Showing the layers exposed to a normal load of 45 N.

**Figure 9:**
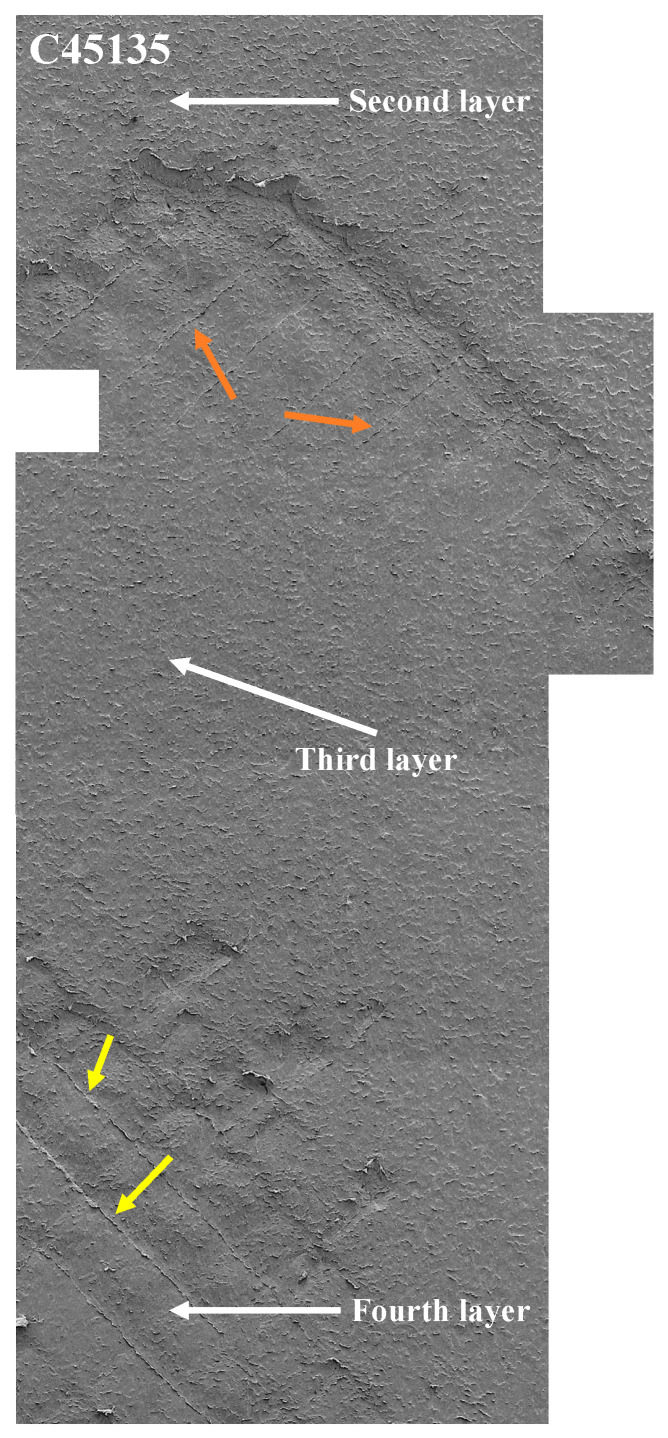
SEM images of sample C45135 show abrasive wear in an enlarged region of the total worn area, with a normal load of 130 N.

The appearance of new layers was most pronounced in sample C900, followed by C45135, under all normal loads. In these cases, alternating the printing direction within each layer reduces the continuity of the deposited filaments, potentially decreasing interlayer cohesion and increasing susceptibility to wear under higher loads. Figure 8 and Figure 9 illustrate the effect of abrasive wear in enlarged regions of the wear tracks, where the detachment of material and exposure of up to four layers are visible. In Figure 8, material removal reveals the printing direction marks, which are indicated with colored arrows, while Figure 9 shows the difference between samples C0 and C900 at 45 N. In this figure, the tearing of filaments along the wear direction is evident in C900, in contrast with the stronger bonding observed in C0 between deposited layers and filaments.

These results indicate that varying only the printing direction generates distinctive inter-filament spacing distributions and differences in interlaminar adhesion, which is directly related to the progressive detachment of multiple layers observed under abrasive wear conditions. This finding suggests that, even without modifying infill density or adding reinforcements, the printing direction can significantly modify the morphology and, consequently, the tribological behavior.

**Figure 10:**
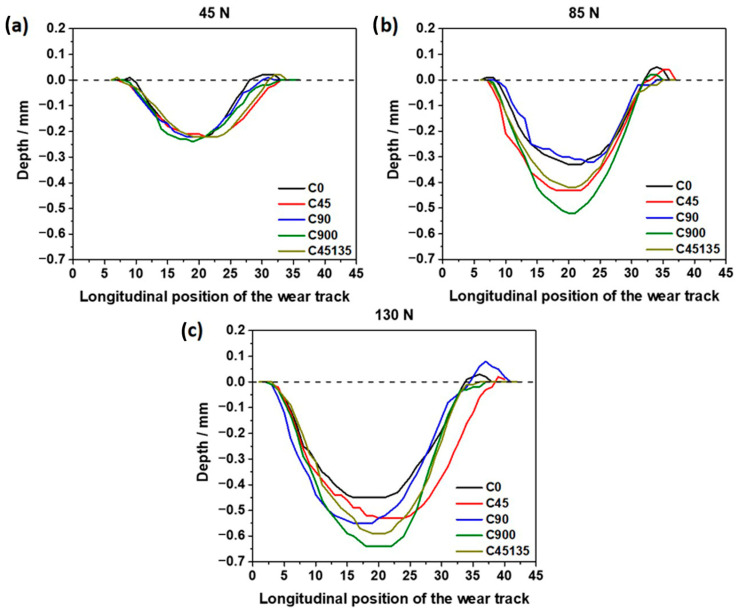
Two-dimensional wear profile for each normal load.

Figure 10 presents the 2D wear profiles of all samples under different normal loads. As the normal load increases, a significant increase in the wear depth is observed, especially in samples C900 and C45135, where the highest depths are recorded. This result is consistent with previously reported mass-loss and worn-area data, confirming that alternating-direction orientations exhibit lower resistance to abrasive wear [40]. A relevant aspect is the accumulation of material (pile-up) at the end of the wear profile, indicating a more pronounced plastic deformation due to material displacement caused by abrasion, particularly under loads of 85 N and 130 N. This behavior suggests that, as the load increases, the structural discontinuities between layers (observed in the SEM images in Figure 8) amplify the effects of wear, favoring material removal. Comparing the C90 and C900 samples reveals a clear difference in the depth and width of the wear profile, further supporting the negative influence of alternating-direction printing configurations on filament cohesion. This difference also correlates with the asymmetry in some wear profiles, where the exposure of new layers by wear generates irregularities in the profile geometry [41,42].

**Figure 11:**
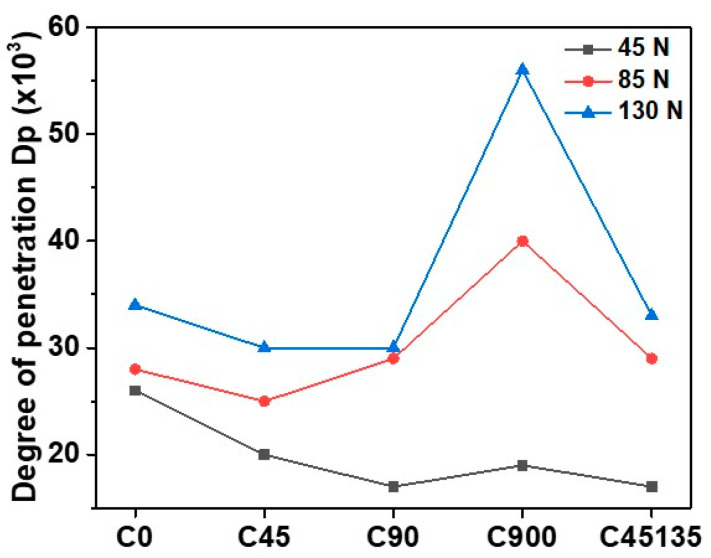
Penetration degree depends on the normal loads.

Figure 11 presents the Dp of the samples as a function of the applied normal load. The results clearly show that sample C900 exhibits the highest Dp values under loads of 85 N and 130 N, indicating a higher plastic deformation capacity and, consequently, easier layer detachment during the wear process. This behavior is directly related to the alternating printing direction in sample C900, which decreases interlaminar cohesion and favors filament separation. On the other hand, sample C0 exhibits lower wear depths at higher loads and Dp values similar to sample C45135, indicating that the d and a parameter in sample C45135 are higher but proportional. This finding aligns with the observations in Figure 6 and Figure 9, where the wear surfaces of sample C0 exhibit less deep and more uniform damage than those of sample C45135. In this case, the printing orientation of sample C0 improves the ability to resist abrasion material, as the direction of the filaments favors a better distribution of stresses.

Sample C45 presents very similar Dp values under loads of 85 N and 130 N, which coincides with the results in Figure 7b, where the exposure of three consecutive layers as a result of wear is observed. This localized exposure amplifies the wear depth and increases the Dp to 85 N.

The printing orientation directly influences the effective contact area between the abrasive particles and the filament surfaces. Samples printed with a single orientation (e.g., C0 and C90) exhibit filaments aligned uniformly along the surface, resulting in a larger continuous contact area per filament, which enhances load distribution and reduces local stresses during abrasion. In contrast, alternating printing directions (e.g., C900 and C45135) create intersecting filament patterns with variable orientations, leading to smaller effective contact areas per filament segment and generating local stress concentrations at filament boundaries. This phenomenon reduces inter-filament cohesion and facilitates progressive material removal under abrasive loading.

### 3.3. Wear Mechanisms

**Figure 12:**
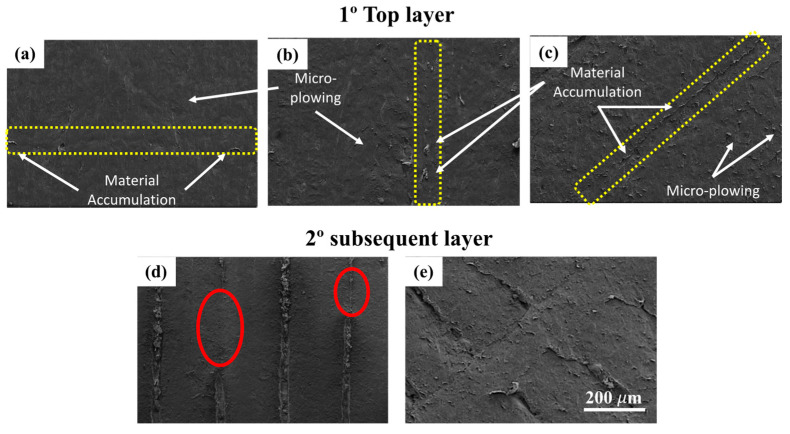
SEM images of worn surfaces of nylon samples under a normal load of 45 N for the five printing orientations. Top row: worn surfaces limited to the top layer for C0 (**a**), C45 (**b**), and C90 (**c**), and Bottom row: exposure of the subsequent layer in C900 (**d**) and C45135 (**e**).

**Figure 13:**
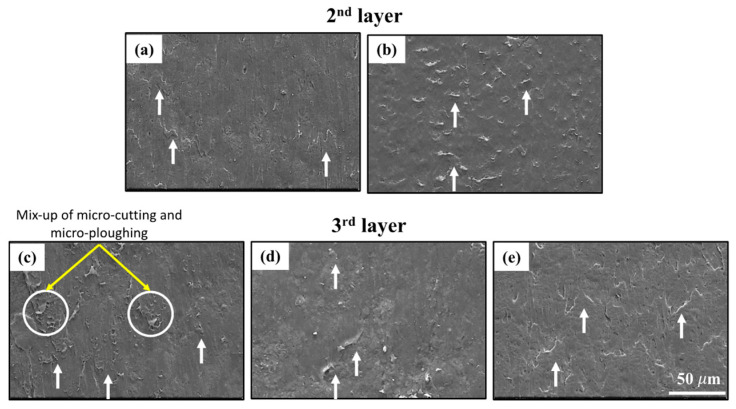
SEM images of the worn surfaces of nylon samples subjected to a normal load of 85 N for the different printing orientations: (**a**) C0, (**b**) C90, (**c**) C45, (**d**) C900, and (**e**) C45135. Second and third layers are exposed depending on the printing orientation.

**Figure 14:**
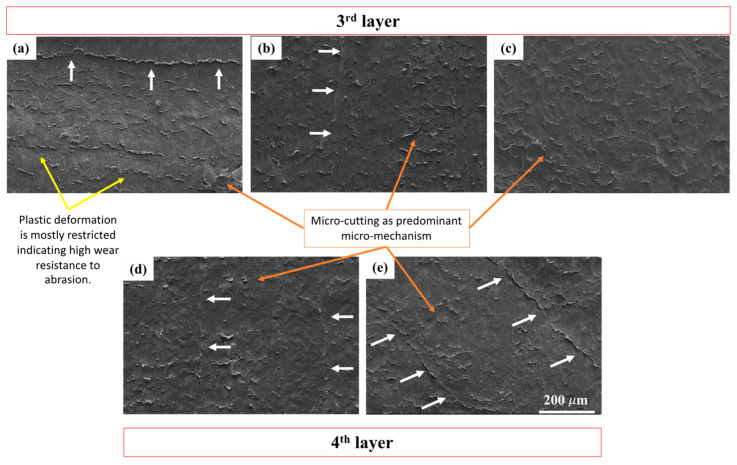
SEM images of the worn surfaces of nylon samples subjected to a normal load of 130 N for the different printing orientations: (**a**) C0, (**b**) C90, (**c**) C45, (**d**) C900, and (**e**) C45135.

**Figure 15:**
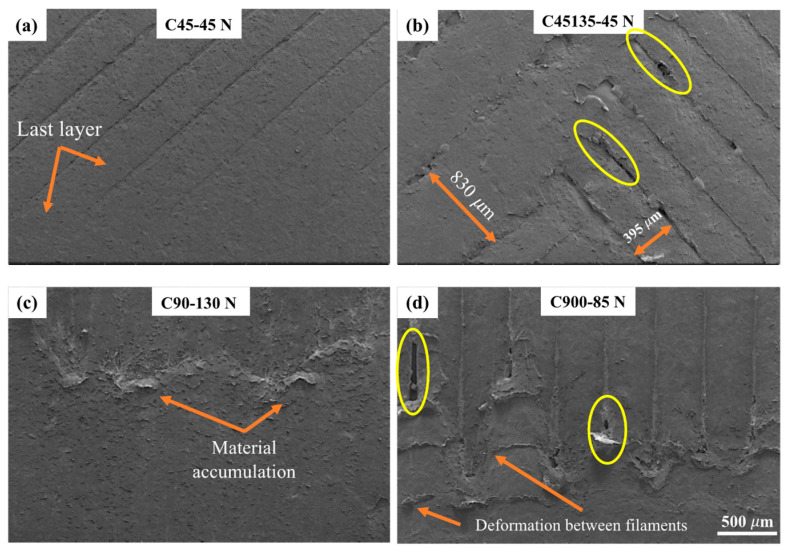
SEM images of different nylon samples under various normal loads: (**a**) C45 at 45 N, (**b**) C45135 at 45 N, (**c**) C90 at 130 N, and (**d**) C900 at 85 N. Orange arrows indicate the exposed layer, material accumulation, and deformation between filaments. Yellow circles highlight separations and discontinuities at the filament junctions, while in (**b**) the spacing between deposited filaments is also indicated.

**Figure 16:**
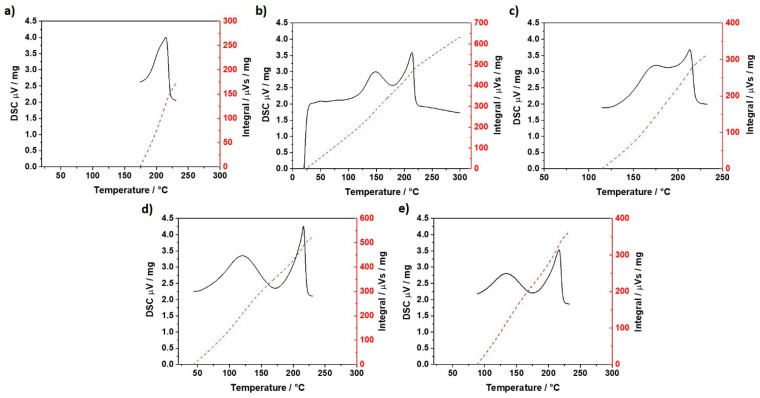
DSC results (**a**) as-received Nylon, (**b**) C0 exposed to abrasion, (**c**) C0 not exposed to abrasion, (**d**) C45135 exposed to abrasion, (**e**) C45135 not exposed to abrasion.

The worn surfaces in the central regions of the samples were analyzed using SEM to understand the material-removal mechanism. Figure 12, Figure 13, Figure 14, Figure 15 and Figure 16 show the evolution of abrasive wear in the samples under different loading conditions. In all cases, both micro-plowing and micro-cutting were identified as the primary microwear mechanisms, consistent with descriptions in the literature [43].

Figure 12 shows SEM images of the worn surfaces of nylon samples subjected to a normal load of 45 N. The top layer presents the surfaces of samples C0 (a), C45 (b), and C90 (c). Here wear is limited to the first layer (top layer). In these cases, the predominant micro-mechanism is micro-plowing. Plastic deformation marks are aligned with the direction of the abrasive particle flow. Material accumulation between adjacent filaments is also observed, as indicated by the white arrows. The yellow dashed lines highlight filament junctions, where material accumulation is also present. This reinforces the presence of micro-plowing under this load condition.

In contrast, samples C900 (d) and C45135 (e) reveal the exposure of the second layer (subsequent layer), indicating that wear has removed the top layer. In these images, the deposition directions of the filaments are more clearly distinguishable, along with less material accumulation between them compared to the other samples. Areas where the filament junctions cannot be identified due to plastic deformation are also visible, marked with a red ellipse. Specifically, in sample C45135, material accumulation is observed both between the filaments of the subsequent layer and partially from the top layer, consistent with the distribution of worn areas shown in Figure 7.

These observations align with the printing orientations schematized in Figure 5, confirming that the micro-mechanism of micro-plowing is predominant across all orientations at this load, although samples C900 and C45135 exhibited greater mass loss and exposure of the subsequent layer.

As shown in Figure 13, the SEM images display the worn surfaces of the nylon samples subjected to a normal load of 85 N for the different printing orientations. The layer exposure process is progressive at this load, with the exposure of the second and third layers. As the top layer detaches, the severity of wear tends to slow down, as part of the abrasive action is consumed in removing the surface layer before significantly affecting the underlying material. As a result, a lower cumulative wear severity than expected for this load is observed, accompanied by greater plastic deformations and a more uniform worn surface, with more visible material accumulations, indicated by white arrows. Under these conditions, wear progresses towards a transition stage from the micro-mechanism of micro-plowing to micro-cutting, evidenced by the presence of regions with plastic deformation oriented in the wear direction.

In particular, the C90 sample shows cavities generated by the penetration of abrasive particles, which cause localized surface deformations. This behavior suggests that the abrasive interaction at this load favors material accumulation as a micro-mechanism of transition, modulating the progression of wear across layers.

Additionally, the C45 and C45135 samples exhibit similar wear profiles and penetration depths (see Figure 10 and Figure 11). However, SEM images reveal greater wear severity in C45. C45 has broader zones of material accumulation and a more pronounced tendency toward micro-cutting. This might relate to both samples sharing the same printing direction in the third layer. This could contribute to greater surface wear and a faster transition toward mechanisms dominated by micro-cutting.

Figure 14 shows the SEM images of the nylon samples tested under a normal load of 130 N. In most samples, the formation of chips along the junctions between deposited filaments is observed, indicated by white arrows. These junctions act as critical regions for the initiation of abrasive wear, consistent with the observations previously made at 45 N and 85 N. Additionally, chip formations are identified along the filaments deposited in the direction of abrasive flow, reinforcing the role of filament orientation in guiding the wear process.

At this load, the micro-mechanism of micro-cutting is established as the predominant wear mechanism, driven by the increase in effective contact area and the low cohesion between layers, both of which are directly related to the printing orientation.

The C0, C45, and C90 samples exhibit exposure of the third layer, whereas C900 and C45135 reach the fourth layer, reflecting a deeper progression of wear in these latter orientations. Despite being at the third layer, the C0 sample still allows for clear identification of filament junctions and displays plastic deformations aligned horizontally, corresponding to the printing direction. This behavior suggests a greater resistance to abrasive wear in C0, likely due to the more homogeneous and continuous arrangement of the filaments, which helps to limit damage propagation compared to the other orientations.

A typical wear scar obtained from the entry and exit zones is shown in Figure 15. The wear scar exhibits three distinct zones: an entry zone where the abrasive particles first contact the sample, a central zone where the particles may roll and slide, and an exit zone where the abrasive particles leave the surface.

In the entry zone, the abrasive particles have low contact with both the rubber wheel and the sample surface, generating minimal stresses in the contact region. These stresses are sufficient to wear the top layer without exposing the subsequent layer (Figure 15a). In contrast, in the sample with two printing directions, the wear track area increases, leading to greater exposure of the filaments (Figure 15b). In this case, the filaments are gradually exposed to the abrasive environment, accompanied by plastic deformation in the top layer, where an increase of 48% in filament width is observed in the first layer compared to the underlying filament (second layer). The exposed filaments detach due to insufficient cohesion between filaments deposited in the same direction, as indicated by the yellow circles.

In the exit zone, material is displaced at the interlayer transition. This leads to material accumulation mainly on the surface filaments (Figure 15c). A larger separation between filaments along the sliding direction is observed, and plastic deformation perpendicular to the sliding direction is observed in the top layer (Figure 15d). The ductile nature of nylon and low cohesion between filaments promote the formation of separations parallel to the sliding direction. Repetitive plowing of the material results in greater mass loss due to more severe damage, particularly in the C900 sample.

These findings highlight the importance of controlling the printing direction as a critical parameter in the design of 3D-printed parts intended for abrasive wear applications. The correlation between layer arrangement, effective contact area, and wear mechanisms provides a basis for optimizing the FDM printing process and enhancing wear resistance.

### 3.4. Thermal Analysis

**Figure 17:**
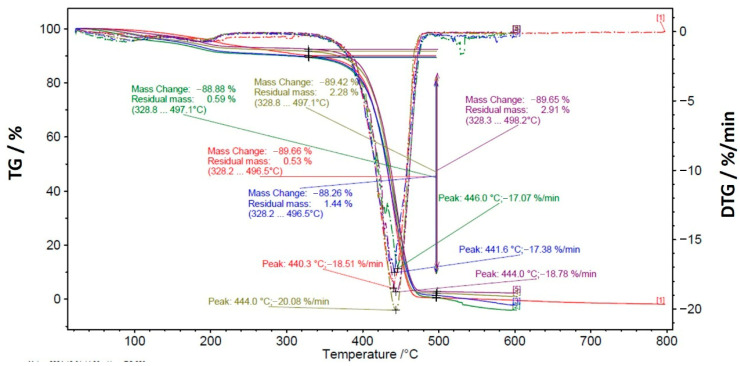
TGA/DTGA results corresponding to: as-received Nylon (red), C0 exposed to abrasion (blue), C0 not exposed to abrasion (purple), C45135 abrasion (green), and C45135 not exposed to abrasion (brown).

Figure 16 and Figure 17 present the thermal analysis of the materials described in this section. Each Figure includes the DSC integral curve. This curve is calculated as the area under the heat flow curve and provides a quantitative measure of the total enthalpy change associated with melting. The peak temperature, T_s_, observed in the DSC results, corresponds to the maximum point of the endothermic peak. This point indicates the temperature at which the material undergoes melting.

Figure 17 shows the TGA. TGA results did not reveal significant differences in mass loss among the tested samples. The peak temperature associated with the decomposition occurred at approximately 440 °C (T_d_) for all samples. At around 500 °C, the nylon mass was completely transformed to the gaseous phase. This suggests that the printing process and wear conditions do not alter the overall thermal stability or chemical composition of the nylon.

Table 3 summarizes the parameters extracted from the DSC. The as-received nylon filament has a T_s_ of 214 °C (T_s2_). The printed samples, regardless of wear exposure, have a T_s_ close to 214 °C (T_s2_). However, unlike the as-received filament, the printed samples presented a second T_s_ at lower temperatures (T_s1_). This ranged from 120 °C (C45135 exposed to abrasion) to 170 °C (C0 not exposed to abrasion). According to Cuiffo et al. [27], the double-peak melting temperature corresponds to the formation of two distinct crystalline phases. One of these phases has a melting temperature lower than that of the virgin Nylon. A decrease in the melting temperature (T_s1_) due to abrasion was also observed for samples printed with both the C0 and C45135. Here, T_s1_ shifted from 170 °C to 145.8 °C and from 133.0 °C to 120.1 °C, respectively.

The enthalpy of printed Nylon associated with the melting process, regardless of exposure to abrasion, was higher than that of the as-received Nylon, with increases ranging from 40% (C0 exposed to abrasion) to 144% (C45135 exposed to abrasion).

The observations made in this section suggest that thermal history mainly induce structural changes, possibly through partial crystallization or molecular rearrangement, which can influence both the thermal stability and mechanical performance of the material.

The printing pattern of Nylon can influence the material enthalpy, particularly under abrasion, due to factors such as thermal history. During the 3D printing process, parameters like extrusion temperature and printing speed significantly impact the crystallinity of polymer-based samples. Higher bed temperatures and optimized printing speeds can enhance crystallinity, resulting in changes to the material thermal properties, including enthalpy [28]. Moreover, studies have shown that different printing patterns can lead to varying degrees of crystallinity and porosity, which in turn affect the material’s mechanical and thermal properties [29]. Additionally, post-printing heat treatments have been shown to improve wear properties by optimizing crystallinity. Such treatments can transform the crystalline structure, thereby affecting the enthalpy of the printed material [29]. Our results are not conclusive regarding the effect of mechanical stress, such as that introduced by abrasion, on the crystallinity of printed nylon. For the C0 pattern, the enthalpy showed no significant difference between samples exposed to abrasion and those that were not. In contrast, the C45135 pattern exhibited a 66% increase in enthalpy when comparing abraded samples to their non-abraded counterparts. Further work is required to establish whether abrasion may promote additional crystallization or disrupt existing molecular structures, thereby leading to variations in enthalpy.

It is important to note that the melting temperatures observed by DSC (T_s1_ and T_s2_) and the decomposition temperature obtained by TGA correspond to different thermal phenomena and are not directly related. While T_s1_ and T_s2_ are melting transitions associated with the degree of crystallinity and structural reorganization of the polymer chains, the decomposition temperature (~440 °C for all samples) marks the onset of the chemical degradation of nylon, involving mass loss and the release of volatiles, with complete conversion to the gaseous phase occurring near 500 °C. The similar thermal stability among the samples indicates that the structural modifications induced by thermal history and mechanical stress—evidenced by changes in T_s1_, T_s2_, and enthalpy—do not compromise the material decomposition temperature. In other words, variations in crystallinity affect the morphology and mechanical behavior but do not reduce the overall resistance of the polymer to thermal degradation.

## 4. Conclusions

This study investigated the effect of printing orientation on the abrasive wear response and thermal behavior of nylon parts manufactured by Fused Deposition Modeling (FDM).

This study demonstrates that printing orientation has a direct and qualitative influence on the abrasive wear response of FDM-printed nylon. Alternating filament orientations produced higher material loss, earlier onset of micro-cutting, and exposure of up to four printed layers under 130 N (Average worn, C900 = 60.6 mm^2^), evidencing lower interlayer cohesion. In contrast, uniformly oriented samples consistently showed milder damage, fewer exposed layers (≤3), and more stable adhesion, confirming a qualitative improvement in wear resistance due to directional filament alignment.

SEM observations and penetration depth measurements revealed that staggered orientations promote earlier transitions to micro-cutting wear mechanisms, accelerating severe wear and mass loss. In contrast, uniform orientations enable a more homogeneous stress distribution, limiting plastic deformation and layer detachment.

Thermal analysis revealed that abrasion reduces the lower-temperature melting peak (Ts1) and increases melting enthalpy by up to 144%, indicating orientation-dependent changes in crystallinity driven by mechanical loading and thermal history. These results qualitatively reinforce that printing orientation governs both the structural integrity and the functional durability of nylon under abrasive conditions.

Overall, optimizing printing orientation emerges as an effective and non-intrusive design variable to enhance the tribological performance of FDM-fabricated nylon components.

## Figures and Tables

**Table 1:** Testing conditions for abrasion wear.

Parameter	Description
Abrasive Type	SiO_2_
Abrasive Size	50 mesh (212–300 μm)
Repeatability	3
Rubber Wheel Speed	200 rpm
Normal Load	45, 85, and 130 N
Abrasive Mass Flow Rate	311 g min^−1^
Test Temperature	25 °C ± 2 °C
Test Duration	10 min

**Table 2:** Average worn area (mm^2^) and standard deviation of each exposed layer for nylon samples printed with different directions under normal loads of 45, 85, and 130 N.

	45 N		85 N		
Printing Direction	First Layer	Second Layer	First Layer	Second Layer	Third Layer
C0	243.1 ± 10.48	-	375.0 ± 6.01	148.8 ± 3.46	-
C45	294.1 ± 11.39	-	402.8 ± 13.36	205.7 ± 22.93	115.1 ± 12.81
C90	298.2 ± 3.34	-	331.3 ± 17.05	75.4 ± 5.56	-
C900	278.7 ± 2.89	68.6 ± 2.12	403.7 ± 5.62	234.9 ± 2.45	125.1 ± 1.22
C45135	29,815 ± 21.09	27.4 ± 0.87	368.2 ± 14.93	110.7 ± 1.86	41.8 ± 0.45
	130 N				
Printing direction	First Layer	Second layer	Third layer	Fourth layer	
C0	441.0 ± 8.23	221.4 ± 15.11	91.5 ± 9.98	-	
C45	436.7 ± 10.72	269.6 ± 21.19	141.2 ± 5.48	-	
C90	431.4 ± 9.71	278.3 ± 4.39	106.1 ± 3.42	-	
C900	463.5 ± 18.51	260.2 ± 4.81	158.0 ± 21.45	60.6 ± 0.93	
C45135	432.1 ± 14.01	227.5 ± 7.22	112.6 ± 3.29	24.1 ± 4.68	

**Table 3:** Extracted parameters from DSC for each sample. T_i_: Initial temperature; T_f_: Final temperature; T_s1_: First melting temperature; T_s2_: Second melting temperature.

Material	T_i_ °C	T_f_ °C	T_s1_ °C	T_s2_ °C	Enthalpy mVs/mg	T_d_ °C
as-received Nylon	174.1	224.5	-	214.3	237.6	440
C0 pattern not exposed to abrasion	125.6	232.1	170	212.8	511.1	
C0 pattern exposed to abrasion	109.1	230.4	145.1	214.1	486.4	
C45135 pattern not exposed to abrasion	89.3	225.5	133.0	216.4	332.4	
C45135 pattern exposed to abrasion	52.2	225.3	120.1	215.9	580.7	

## Data Availability

The original contributions presented in this study are included in the article. Further inquiries can be directed to the corresponding authors.
